# Correlation between semen quality and the seminal biochemical markers: alpha-glucosidase, fructose, and zinc in infertile men compared with a normal population of men

**DOI:** 10.3389/fendo.2025.1583272

**Published:** 2025-08-08

**Authors:** Chaymae Rochdi, Fahd A. Nasr, Amal Elrherabi, Ibtissam Bellajdel, Hafsa Taheri, Mohamed Bouhrim, Mohammed Al-zharani, Ashraf Ahmed Qurtam, Hanane Saadi, Ahmed Mimouni, Mohammed Choukri

**Affiliations:** ^1^ Maternal-Child and Mental Health Research Laboratory, Faculty of Medicine and Pharmacy, Mohammed First University, Oujda, Morocco; ^2^ Medically Assisted Procreation Unit, Central Laboratory, Mohammed VI University Hospital Center, Oujda, Morocco; ^3^ Biology Department, College of Science, Imam Mohammad Ibn Saud Islamic University (IMSIU), Riyadh, Saudi Arabia; ^4^ Laboratory of Bioresources, Biotechnology, Ethnopharmacology and Health, Faculty of Sciences, University Mohammed, Oujda, Morocco; ^5^ Laboratory of Biological Engineering, Team of Functional and Pathological Biology, Faculty of Sciences and Techniques Beni Mellal, University Sultan Moulay Slimane, Beni Mellal, Morocco

**Keywords:** zinc, fructose, male infertility, neutral alpha-glucosidase, seminal plasma, sperm parameters

## Abstract

**Introduction:**

The male reproductive tract's accessory glands produce seminal biochemical markers that can help diagnose reproductive disorders and assess male fertility. This study evaluated the relationship between seminal biochemical components and sperm parameters in 150 men, including 20 normospermic individuals and 130 infertile patients classified into oligozoospermia, azoospermia, asthenozoospermia, teratozoospermia, and oligoasthenoteratozoospermia (OAT) groups.

**Methods:**

The patients underwent semen analysis and measurements of fructose, neutral alpha-glucosidase (NAG) and zinc in seminal plasma.

**Results and discussions:**

The level of fructose was significantly decreased in asthenozoospermic and increased in oligoasthenoteratozoospermic (OAT) men. It was significantly correlated with semen volume, sperm concentration, progressive motility and morphology. Seminal neutral -glucosidase and zinc levels were found significantly reduced in azoospermic and OAT patients. The seminal NAG levels were significantly correlated with semen volume and progressive motility. For zinc level, the concentration was significantly correlated with sperm concentration (r = 0.041, p < 0.001).

## 1 Introduction

Infertility is a condition affecting the reproductive system that affects 13% to 18% of the world’s population and is marked by the inability to conceive after 12 months or more of regular, unprotected intercourse (1). The part of men in couple infertility represents more or less 50% (2). Male infertility pathophysiology may be attributed to a sequence of molecular and biochemical processes, the majority of which are manifested by aberrant semen parameters (3). The testicles produce spermatozoa, that are secreted into the lumen of the seminiferous tubules for maturation and progression. Once spermatozoa have fully developed their head and tail, they are released from the cell and transported into the epididymis (4). The epididymis and accessory sex glands are essential for enabling spermatozoa to acquire functionality and maturity necessary for egg fertilization (5). Semen, also known as ejaculate, is the fluid expelled from the penis at the moment of orgasm. It consists of two primary components: a cellular component, which includes spermatozoa, and a non-cellular component, known as seminal plasma (6). Semen is a slightly viscous, whitish, and milky fluid composed primarily of water. It contains various dissolved components, including salts, proteins, fructose, citric acid, and other essential nutrients and enzymes that support sperm function and viability (7). Spermatozoa constitute only about 5 to 10% of semen volume. The seminal vesicles contribute 40 to 80% of semen, supplying fructose for sperm nourishment, prostaglandins, coagulating agents, and bicarbonate to help neutralize the acidic environment of the vaginal tract. The prostate generates 10 to 30% of the seminal plasma (8). Human prostate secretions include significant concentrations of citric acid, acid phosphatase, phospholipids, spermine, and enzymes and proteases that aid in liquefying the seminal coagulum. The bulbourethral (Cowper) glands produce a small amount of seminal fluid (9). Seminal plasma is a combination of various secretions from several male accessory glands: fructose and prostaglandins are sourced from the seminal vesicles; citrate, zinc, and prostate-specific acid phosphatase are produced by the prostate; and NAG, carnitine, and glycerophosphocholine, lipid, carbohydrates, and steroids for the epididymis (8). Currently, NAG, zinc, and fructose levels in seminal plasma are studied to understand the secretory function of male accessory glands and their associations with semen quality. However, the clinical diagnostic value of these markers remains to be fully established (10). Seminal plasma provides an environment that supports sperm motility and survival, but the biochemical components and their physiological roles are not yet completely understood (11). The epididymis is a tightly coiled tube where sperm undergo the final phases of growth and maturation. It plays a crucial role in enabling sperm to acquire progressive motility and the capacity to fertilize an egg. NAG measurement may be useful in assessing epididymal pathology (12). Several studies have reported associations between low seminal NAG levels and conditions such as impaired sperm maturation and genital tract inflammation (13). Conversely, higher NAG levels have been linked to sperm functional properties in some contexts (12). Semen’s pH is maintained by zinc. It has been demonstrated that lower values of these markers serve as a diagnostic tool for assessing the prostate’s secretory dysfunction (14). The seminal fructose has been identified as an energy source for the motility of sperm cells. A decrease in fructose levels is typically associated with obstruction of the ejaculatory ducts or absence of seminal vesicles. This is particularly evident when accompanied by low ejaculate volume in a patient, suggesting ejaculatory duct obstruction, seminal vesicle dysfunction, or hypoplasia, and is often seen in cases of asthenozoospermia (15). The positive relationship between sperm motility and the levels of biochemical markers like fructose, zinc, and NAG remains a topic of discussion (16). Additionally, the sources and possible physiological effects of biochemical components in seminal plasma remain inadequately understood. To our knowledge, this study is the first of its kind conducted in Morocco in this field. Therefore, the aim of the present study was to compare the concentrations of NAG, fructose, and zinc in the seminal plasma of infertile patients and to investigate the relationships between these biochemical markers and semen parameters in infertile Moroccan males, with a view to exploring their potential as supplementary tools alongside the traditional spermiogram.

## 2 Materials and methods

### 2.1 Study population

The study utilized semen samples collected from 150 infertile men aged 28 to 36 years who were undergoing infertility evaluation at the Department of Reproductive Biology (Mohammed VI University Hospital Center of Oujda, Morocco), for the evaluation of infertility between April 2023 and December 2023. Participants currently taking any medication or antioxidant supplements, as well as those experiencing any acute infections, were excluded from the study. The subjects were males visiting fertility clinics who reported an inability to achieve pregnancy for at least one year after marriage, with no evident chronic or acute illnesses.

The samples were classified into three groups based on sperm count: normozoospermic (n=20), oligozoospermic (n=28), and azoospermic (n=32). They were also categorized according to sperm motility into progressively motile and asthenozoospermic (n=22) groups. Additionally, based on sperm morphology, the samples were divided into normal and teratozoospermic (n=18) for analysis. Further, based on several alterations of semen parameters, oligoasthenoteratozoospermic (OAT) (n=30). It should be noted that the normozoospermic group is smaller compared to other groups, as the study was conducted in a fertility clinic where the majority of patients presented with infertility issues. This limitation may affect the statistical power and generalizability of comparisons involving this group.

### 2.2 Study ethics and consent

This study was approved by the Ethics Committee for Biomedical Research (ECBR) of the Faculty of Medicine and Pharmacy of Oujda, Mohammed First University, under the reference number 02/2023. The ethical review process began on January 9, 2023, and final approval was granted on April 4, 2024. All participants were fully informed of the study’s objectives and provided written, informed consent prior to participation.

### 2.3 Semen analysis

The semen samples were obtained by masturbation into a sterile container after a sexual abstinence period of 2 to 3 days. Following 30 minutes of liquefaction, the ejaculates were assessed for several macroscopic characteristics (including pH and volume) and microscopic parameters, such as sperm concentration, progressive motility, viability, and morphology, in accordance with WHO guidelines 2021 (17).

First, the semen samples were homogenized using a Pasteur pipette, and sperm concentration and motility were evaluated with the Computer Assisted Sperm Analyzer (SCA, MICROPTIC, Barcelona, Spain). A 2.5 µL aliquot of the sperm sample was then placed on a standard four-chamber slide (Leja, NL, Nieuw-Vennep, the Netherlands) for analysis. Spermatozoa with both fast and slow progressive motility were counted (A+B), in addition to non-progressive motile sperm (C) and non-motile sperm (D). The assessment of sperm concentration and motility was conducted at a magnification of ×10.

Sperm morphology was evaluated based on Modified David criteria, using the Diff-Quik kit (Dade Behring AG, Dudingen, Switzerland), which includes one fixative stain and two additional stains (A, B). Morphological evaluations were carried out with an oil immersion Nikon microscope (Nikon Company, Tokyo, Japan), with at least 100 sperm cells counted. Strict criteria were followed to categorize men as having normal or abnormal morphology according to the modified David classification.

### 2.4 Normal parameters

The 2021 World Health Organization (WHO) guidelines for human semen examination and processing specify the following reference values: semen volume should be ≥ 1.6 mL; pH should be ≥ 7.2; liquefaction time should be less than 60 minutes; sperm count should be ≥ 16 million/mL; progressive motility should be at least 30%; total motility should be ≥ 40%; vitality should reach ≥ 54%; the proportion of sperm with normal morphology should be ≥ 4%; and the DNA fragmentation index should not exceed 25% (17).

### 2.5 Biochemical assays

Biochemical markers were evaluated to assess the functions of the epididymis (NAG), the prostate (zinc), and the seminal vesicles (fructose). Liquefied semen was centrifuged at 3000g for 15 min at 24°C within 2 h after sampling and seminal plasma was promptly processed for further analysis without undergoing freeze–thaw cycles. The upper layer of seminal plasma was collected to assess biochemical markers. It was carefully decanted and stored at -80°C until analysis, where levels of seminal biomarkers (fructose, zinc, and neutral α-glucosidase) were measured using analytical spectrophotometry.

#### 2.5.1 Fructose assay

Fructose concentration was determined through an enzymatic assay method. The hexokinase decomposes the fructose as fructose-6-phosphoric acid, whereas the latter then changes its structure as glucose-6-phosphoric acid. Glucose-6-glycerol phosphate dehydrogenase catalyzes the glucose-6-phosphoric acid and NAD+ and thus produces the NADH. By monitoring the NADH absorbance at 340nm wavelength, a,d eliminating the effect of glucose over the results, the fructose concentration in the specimens then can be obtained. Fructose concentration in seminal plasma was measured according to kit instructions (SemenAssay Fructose kit at BRED Life Science Technology Inc., China). Fructose concentration was determined by measuring its absorbance at 340 nm.

#### 2.5.2 Zinc assay

Zinc produces Zn-5-Br-PAPS complex under alkaline conditions with 2-(5-bromo-2-pyridylazo)-5-(N-propyl-N-sulfo-propylamide)-phenol (5-Br-PAPS), and the latter has the maximum absorption wavelength at 560nm, where the absorbance is in proportion to the zinc level in semen plasma. Zinc concentration in seminal plasma was measured according to kit instructions (SemenAssay Zinc kit at BRED Life Science Technology Inc., China).

#### 2.5.3 NAG assay

There’s NAG and a small amount of acid alpha-glucosidase in the seminal plasma, and after inhibiting the reaction of acid alpha-glucosidase, the NAG then decomposes the substrate and produces the end product-para-nitrophenol (PNP), which has the maximum absorption wavelength at 405nm, and its absorbance is in proportion to the volume of NAG. The enzyme concentration in seminal plasma was measured according to kit instructions SemenAssay^®^ NAG kit (catalog number: BRED-007) from BRED Life Science Technology Inc., China.

### 2.6 Statistical analysis

Descriptive statistical analyses were conducted using SPSS software, version 9.0 (Statistics Package for Social Sciences Inc., Chicago, IL, USA). The normality of biochemical marker data was assessed using the Shapiro–Wilk test, and the results confirmed that the data followed a normal distribution (p > 0.05 for all variables). Therefore, differences within study groups were analyzed using analysis of variance (ANOVA) followed by Tukey’s Honest Significant Difference (HSD) *post hoc* test. Bivariate correlations between biochemical markers in semen and sperm parameters were assessed through Pearson’s correlation coefficient. A p-value < 0.05 was considered statistically significant.

## 3 Results

**Table 1** presents the general characteristics of the study population. The average age of participants was 31.85 ± 1.07 years, with the majority residing in urban areas. Of the subjects, 20 were classified as normozoospermic, 28 as oligozoospermic, 32 as azoospermic, 22 as asthenozoospermic, 18 as teratozoospermic, and the remaining 30 as having oligoasthenoteratozoospermia (OAT).

**Table 1:** Seminal characteristics (values mean ± SD) for each group of patients.

Parameters	Normozoospermic	Oligozoospermic	Azoospermic	Asthenozoospermic	Teratozoospermic	OAT
Number	20	28	32	22	18	30
Age (years)	30.24 ± 2.67	32.40 ± 1.34	31.01 ± 0.62	32.22 ± 1.91	30.76 ± 1.05	32.46 ± 3.84
Volume (ml)	2.5 ± 0.52	2.8 ± 1.12	3.3 ± 0.63	2.6 ± 0.94	2.5 ± 1.39	3.7 ± 0.12
Concentration (106/ml)	22.1 ± 2.71	7.1 ± 1.42	–	18.43 ± 1.10	25.23 ± 2.06	6.21 ± 2.98
Progressive motility (a+b) (%)	42.33 ± 1.57	38.11 ± 2.04	–	21.23 ± 2.90	36.18 ± 1.13	15.04 ± 2.63
Viability (%)	72 ± 3.11	64 ± 2.65	–	48 ± 1.11	64 ± 2.07	42 ± 3.15
Morphology (%)	18.41 ± 1.73	15.90 ± 2.34	–	12.13 ± 2.56	3.32 ± 2.63	2.35 ± 1.08

**Table 1** also presents seminal characteristics based on sperm count classifications. Normozoospermic individuals showed significantly higher values in both total progressive motility and normal sperm morphology.

**Table 2** provides an overview of the biochemical marker levels across various subgroups, while **Table 3** compiles the results of their comparisons using ANOVA analysis.

**Table 2:** Descriptive statistics of biochemical parameters expressed as mean ± SD analyzed in the study.

Patients groups	Zinc (µmol/ejaculate)	Fructose (µmol/ejaculate)	Neutral α- Glucosidase (mU/ejaculate)
Normospermic (n=20)	6.4 ± 0.2	16.4 ± 1.3	109.3 ± 5.84
Oligozoospermic (n=28)	5.3 ± 1.6	15.9 ± 2.8	98.7 ± 3.43
Azoospermic (n=32)	1.8 ± 2.7	17.2 ± 3.4	79.2 ± 6.31
Asthenozoospermic (n=22)	5.2 ± 1.9	9.8 ± 2.9	87.1 ± 3.72
Teratozoospermic (n=18)	6.2 ± 3.1	16.8 ± 1.8	102.6 ± 3.97
OAT (n=30)	1.2 ± 2.4	18.2 ± 2.3	93.2 ± 6.72

**Table 3:** ANOVA analysis of biochemical parameters with Tukey HSD *post hoc* test comparing normozoospermic and other groups.

Biochemical Marker	N vs O	N vs Az	N vs As	N vs T	N vs OAT
Zinc	0.743	0.052	0.134	0.903	0.854
Fructose	0.173	0.052	0.083	0.432	0.595
Neutra α-glucosidase	0.274	0.002	1	0.321	0.094

N, Normozoospermic; O, Oligozoospermic; Az, Azoospermic; As, Asthenozoospermic; T, Teratozoospermic.

*Significant at the level p < 0.05.

Significant reductions in seminal NAG and zinc levels were observed in azoospermic and oligospermic patients, with zinc levels decreased across all infertile groups. Seminal fructose levels showed a slight increase in oligospermic patients and those with abnormal sperm morphology but were lower in azoospermic and asthenozoospermic groups, as shown in **Table 2**.

**Table 4** shows that there were statistically significant correlations between certain biochemical markers and semen parameters. NAG concentration (a marker of epididymal function) showed a strong positive correlation with semen volume (r = 0.652, P < 0.001) and a weaker but significant positive correlation with progressive motility (r = 0.102, P < 0.05). Fructose concentration (from seminal vesicles) was positively correlated with semen volume (r = 0.382, P < 0.05) and abnormal sperm morphology (r = 0.357, P < 0.001), but negatively correlated with sperm concentration (r = -0.065, P < 0.05). Zinc concentration (from the prostate) showed a negative correlation with progressive motility (r = -0.063, P < 0.05), while its correlation with semen volume was not significant (r = -0.074, ns). These associations suggest a link between glandular secretory function and key semen parameters relevant to fertility.

**Table 4:** Pearson correlation coefficients (r) between semen parameters and biochemical markers in seminal plasma.

Biochemical Marker	Volume (ml)	Sperm Concentration (×10^6^/ml)	Progressive Motility (%)	Abnormal Morphology (%)
Zinc (µmol/ejaculate)	-0.074^ns^	0.041^a^	-0.063^b^	-0.011^b^
Fructose (µmol/ejaculate)	0.382^b^	-0.065^a^	-0.095^b^	0.357^a^
Neutral α-glucosidase (mU/ejaculate)	0.652^a^	0.743^b^	0.102^b^	0.093^a^

Significance levels: *a* p < 0.001; *b* p < 0.05; ns, not significant.

a Significant at the level P< 0.001.

b Significant at the level P< 0.05.

^ns^Nonsignificant.

## 4 Discussion

Reproductive success depends on sufficient sperm motility, concentration, and morphology, all known to be impacted by deteriorating semen quality (18). The movement of spermatozoa is considered one of the most essential indicators of male fertility, as it significantly enhances the likelihood of successful fertilization (2). This is the first study linking the amounts of zinc, fructose, and NAG in seminal plasma with characteristics of semen involving samples from both fertile and infertile men in Morocco. This study aimed to determine whether biochemical parameters in seminal plasma are altered in Moroccan infertile men presenting with either normozoospermia, oligozoospermia, azoospermia, asthenozoospermia, teratozoospermia, and oligoasthenoteratozoospermia (OAT). Seminal plasma has been investigated in great detail throughout the last seven decades (9). Nonetheless, there is a dearth of documentation about the comparison of biochemical markers in seminal plasma (10, 15). Seminal plasma is a mixture of different secretions from multiple male accessory glands: fructose and prostaglandins for seminal vesicles; citrate, zinc, and prostate-specific acid phosphatase for the prostate; and NAG, carnitine, glycerophosphocholine, lipid, carbohydrates, and steroids for the epididymis (19). At present, levels of NAG, zinc, and fructose in seminal plasma are measured in clinical andrology laboratories to assess the secretion function of male accessory glands (8). The seminal plasma fructose content can be influenced using a range of parameters, including blood glucose levels, frequency of ejaculation, and nutritional condition, in addition to the androgenic regulation of fructose secretion (20). Because seminal vesicles make up 60% of the overall volume of semen, a reduced volume of ejaculate may indicate a decreased secretory activity of this gland (9). In particular, the reduced fructose content observed in asthenozoospermic patients may indicate dysfunction of the seminal vesicles or reduced energy substrate availability for sperm motility. These findings support the clinical relevance of fructose as a functional marker of seminal vesicle activity and may assist in assessing specific underlying causes in patients with isolated motility issues. According to our findings, there is a negative correlation between sperm count and fructose concentration and a positive correlation with semen volume. Fructose in seminal plasma is considered the key energy source for sperm metabolism and motility *in vitro* (21). In asthenozoospermic men, the fructose content was much lower, whereas, in asthenoteratozoospermics, it was higher (22). Fructose, primarily produced by the seminal vesicles, is a vital energy source for sperm motility and metabolism. Its presence is crucial for sperm survival and function within the female reproductive tract. Low seminal fructose levels can indicate seminal vesicle dysfunction, ejaculatory duct obstruction, or hormonal imbalances, all of which can contribute to male infertility. Conversely, some studies suggest a negative correlation between high fructose levels and sperm concentration or motility, as sperm consume fructose for energy (23). Seminal fructose levels and the percentage of motile sperm were shown to positively correlate by Abdellah et al. (16). The elevated fructose levels in asthenoteratozoospermic men may be attributed to its reduced utilization by spermatozoa with morphological defects (22). Increased seminal fructose levels were observed in OAT and azoospermic patients (23). This may be due to the decreased concentration and activity, which leads to lower consumption of the synthesized fructose. Conversely, seminal fructose levels were reduced in oligospermic and asthenozoospermic patients (22). Our findings revealed no significant difference in the levels of fructose and zinc in the seminal plasma of Moroccan patients when compared to the control group. NAG is predominantly secreted by the epididymis, mainly in the corpus and cauda regions. It plays a role in breaking down carbohydrates, providing energy for sperm metabolism and motility. Low levels of α-GLUC in semen can indicate epididymal dysfunction or obstruction, which can impair sperm maturation and contribute to male infertility. Therefore, α-GLUC activity is considered a reliable marker for assessing epididymal function and patency (24). It is an accurate and noteworthy indicator for determining epididymal potency (25). It has been demonstrated that the epididymis is essential for sperm maturation, particularly for the development of motility and/or the ability to fertilize (26). They discovered that the aberrant spermatograms and NAG activity were inversely correlated (27). These findings are in good agreement with those of Said et al. (2009), who demonstrated an inverse relationship between sperm count and seminal fructose content. Their study also reported a considerable reduction in seminal neutral α-glucosidase (NAG) levels among infertile patients, which aligns with our observation of significantly lower NAG levels in azoospermic individuals. Since NAG is secreted by the epididymis and regulated by testosterone, its decline may reflect impaired epididymal function and hormonal imbalance, further supporting its role as a diagnostic biomarker in male infertility (10). The significant decline in NAG levels in azoospermic men suggests its potential as a non-invasive biochemical marker to aid in differentiating between obstructive and non-obstructive azoospermia. Given that NAG is secreted by the epididymis and reflects its functional status, measuring its levels can support clinicians in evaluating the cause of azoospermia and guide further diagnostic or therapeutic decisions. The significant reduction in NAG levels observed in azoospermic patients may be attributed to obstruction of the initial segment of the ejaculatory duct near the epididymis (28). Abdellah et al. found that NAG activity was significantly higher in patients with asthenozoospermia compared to other groups. In contrast, our study identified a significant correlation between NAG levels and two factors: semen volume and seminal pH (16). This is consistent with the findings of Henkel et al., who reported a significant correlation between seminal NAG activity and sperm concentration, ejaculate volume, and pH (29). Similarly, previous studies have shown that elevated NAG levels are associated with stronger binding in the sperm-zona pellucida interaction and a higher likelihood of success following intrauterine insemination (30). It has been proposed that NAG activity may serve as a predictor for the outcome of *in vitro* fertilization (IVF) (31). Zinc synthesis and secretion play a crucial physiological role in the functioning of the prostate gland (32). Zinc plays a crucial role in prostate function by accumulating in prostate cells at high concentrations. This high zinc level inhibits mitochondrial aconitase, leading to citrate accumulation, a key component of prostatic fluid. Additionally, zinc acts as a tumor suppressor by inducing apoptosis (programmed cell death) in prostate cells and inhibiting their proliferation and growth. It also helps maintain DNA integrity and can reduce inflammation (33). In our study, the zinc concentration exhibited a negative correlation with seminal pH, with the acidity primarily influencing the pH level. Seminal zinc levels were slightly lower in OAT patients and significantly reduced in azoospermic and oligospermic patients (33). As seminal zinc is primarily secreted by the prostate gland, any partial or complete blockage of the ejaculatory ducts could lead to a reduction in its levels in semen (34). Impaired testosterone secretion and infections of the accessory glands can also result in lower seminal zinc levels (35). Overall, both zinc concentrations and NAG activity were found to decrease significantly as seminal abnormalities increased, particularly in infertile azoospermic patients (36). NAG levels are regarded as a key marker due to their role in sperm maturation and the development of motility. A weak positive correlation was found between NAG, zinc, and fructose in infertile patients with azoospermia, as these markers are influenced by the secretory functions of the accessory glands, a relationship that was most apparent in azoospermic individuals. Although specific diagnostic thresholds for seminal plasma markers such as NAG and fructose are not yet universally established, our findings point toward their potential clinical applicability. The observed patterns in different semen abnormalities underline the importance of establishing population-specific reference ranges and clinical cut-off values, particularly for the Moroccan male population. Further large-scale studies are warranted to validate these markers and define their sensitivity and specificity for different types of male infertility.

One limitation of this study is the standardized 2–3 day abstinence period prior to semen collection. Although this duration aligns with common clinical guidelines, variations in abstinence time across different studies may influence seminal biochemical marker concentrations such as zinc, fructose, and NAG. Shorter or longer abstinence intervals can affect semen volume, sperm concentration, and accessory gland secretions, potentially impacting the comparability of results with other research. Future studies might consider exploring the effect of varying abstinence periods on seminal biochemical parameters to better standardize and interpret these markers in the context of male infertility.

## 5 Conclusions

In conclusion, our findings demonstrate a complex relationship between fructose and both cytological and morphological sperm parameters, while NAG and zinc concentrations are significant determinants primarily for cytological factors. The results suggest that fructose, along with NAG and zinc, serves as sensitive markers of semen quality in men experiencing infertility. Greater attention should be given to the role of seminal vesicles, especially fructose levels in asthenozoospermic and oligoasthenoteratozoospermic patients, and to the exploration of therapeutic approaches for male fertility. Seminal NAG, fructose, and zinc show significant correlations with semen quality and may be useful in assessing male reproductive health.

## Data Availability

The original contributions presented in the study are included in the article/supplementary material. Further inquiries can be directed to the corresponding authors.
